# Role of MicroRNA-21 in Regulating Intracellular Pathways Associated With Phagocytosis in Human Macrophages: An In Vitro Study

**DOI:** 10.7759/cureus.63736

**Published:** 2024-07-03

**Authors:** Natsuki Anada, Yoshinobu Nakayama, Jun Takeshita, Kyoko Kageyama, Hiroatsu Sakamoto, Takahiko Kamibayashi, Yasufumi Nakajima

**Affiliations:** 1 Anesthesiology and Intensive Care Medicine, Kansai Medical University, Hirakata, JPN; 2 Anesthesiology and Critical Care, Faculty of Medicine, Kindai University, Osakasayama, JPN; 3 Anesthesiology and Critical Care, Osaka Women's and Children's Hospital, Osaka, JPN; 4 Outcomes Research Consortium, Cleveland Clinic, Cleveland, USA

**Keywords:** inflammation, intracellular signaling peptides and proteins, macrophage, phagocytosis, mir-21, microrna

## Abstract

Introduction

The efficient clearance of bacteria by macrophages is crucial for the timely resolution of inflammation. In this study, we investigated the role of microRNA-21 (miR-21)-induced phagocytosis and its intracellular signaling pathways in human macrophages *in vitro*.

Methods

Human peripheral blood mononuclear cells (PBMCs) were isolated from whole blood collected from 15 healthy volunteers. Differentiated human macrophages were incubated with lipopolysaccharide (LPS) to determine the time course of changes in phagocytic activity and miR-21 expression. The expression of candidate genes targeted by miR-21 and its downstream effectors was quantitatively assessed. The effects of miR-21 modulation were also examined via transfection with miR-21 mimics and inhibitors.

Results

Incubation of human macrophages with LPS upregulated both phagocytosis and miR-21 expression. Notably, changing miR-21 expression levels using miR-21 mimics or inhibitors led to significant and opposite changes in the expression of its downstream effectors. miR-21 induction in macrophages downregulated PDCD4 and PTEN, promoted the phosphorylation of Akt and the production of the anti-inflammatory cytokine IL-10, and facilitated phagocytosis.

Conclusion

This study directly confirms that LPS upregulates macrophage phagocytosis and miR-21 expression. Elevated miR-21 levels in macrophages enhanced phagocytosis, contributing to an anti-inflammatory phenotype. These findings underscore the importance of miR-21 in resolving inflammation.

## Introduction

MicroRNA-21 (miR-21) are small non-coding RNA molecules that play critical roles in regulating gene expression within cells. miR-21 plays a significant role in various cellular processes and has been extensively studied due to its involvement in various diseases [1]. miR-21 can modulate the activity of immune cells and the expression of cytokines, influencing both innate and adaptive immune responses [2]. However, the exact mechanisms underlying the function of miR-21 remain unclear.

Macrophages are versatile cells essential for both initiating and regulating the innate immune response, as well as linking it to the adaptive immune system. Their functions include phagocytosis, pattern recognition, cytokine production, antigen presentation, production of reactive oxygen and nitrogen species, and tissue repair [3]. The efficient clearance of bacteria by macrophages, known as phagocytosis, is crucial for the timely resolution of inflammation at an injury site. Phagocytosis in macrophages is a highly regulated and dynamic process that involves the coordinated interplay of receptor-mediated signaling, cytoskeletal rearrangement, membrane trafficking, and antimicrobial mechanisms. It plays an important role in both innate immunity, serving as a frontline defense against invading pathogens, and in adaptive immunity through antigen presentation [4,5].

In the present study, we aimed to elucidate the role of miR-21 in human macrophages by investigating whether miR-21 can modulate the phagocytic activity of human macrophages *in vitro*.

## Materials and methods

Ethical approval

With approval from the Review Board for Human Experiments of Kyoto Prefectural University of Medicine (protocol number: ERB-C-288), 15 healthy male volunteers were recruited. The inclusion criteria were as follows: (1) males aged 25-50 years, without any diseases under treatment; and (2) ability of participants to understand and willingness to sign an informed consent document. The exclusion criteria were as follows: (1) persons taking any medications within 2 weeks before participating in the study; (2) blood donation within the last 6 weeks or hemoglobin levels below normal; and (3) active acute illness. Participants could have returned for evaluation at a later date once the acute illness resolved.

Human macrophage differentiation

Human peripheral blood mononuclear cells (PBMCs) were isolated from 20 ml of whole blood using Histopaque®-1077 (Cat. No. 10771, Sigma-Aldrich, St. Louis, MO, USA) according to the manufacturer’s protocol. Briefly, human PBMCs were washed twice with phosphate-buffered saline (PBS), resuspended (5×10^6^ cells ml^-1^) in endotoxin-free Roswell Park Memorial Institute (RPMI) 1640 medium (Gibco, Grand Island, NY, USA) containing 10% human serum, 10 mM HEPES, 2 mM L-glutamine, 1 mM sodium pyruvate, 50 μM 1-mercaptoethanol, 1% streptomycin, 1% penicillin, and 5.5 mM glucose, and cultured for two hours (95% air, 5% CO2; 37°C). Subsequently, the spent medium containing non-adherent cells was discarded, and the adherent monocytes were washed with pre-warmed PBS. Monocytes differentiated into macrophages after 7 days in a 5.5 mM glucose-containing RPMI 1640 medium supplemented with 5 ng ml^-1^ macrophage-colony stimulating factor (M-CSF) (Cat. No. 216-MC-010, R&D Systems, Minneapolis, MN, USA). The medium was changed every two days.

Assessment of macrophage phagocytosis

To assess the phagocytic ability of human macrophages against *Escherichia coli*, fluorescein-isothiocyanate-labeled *E. coli* was provided for ingestion and analyzed using a Vybrant™ Phagocytosis Assay Kit (Cat. No. V6694, Thermo Fisher Scientific, Waltham, MA, USA) according to the manufacturer’s protocol. The cells were washed three times with ice-cold PBS and scanned using a fluorescence plate reader (Tecan, Männedorf, Switzerland) at excitation/emission wavelengths of 485/535 nm. Data were expressed as relative changes from the baseline relative fluorescence units (RFUs).

Real-time polymerase chain reaction

Total RNA was isolated using a miRNeasy Kit (Cat. No. 217084, Qiagen, Hilden, Germany) according to the manufacturer’s instructions. Reverse transcription was performed using a TaqMan™ MicroRNA Reverse Transcription Kit (Cat. No. 4366596, Thermo Fisher Scientific) according to the manufacturer’s protocol. Exogenous non-human cel-miR-39 was spiked into the samples as a control for miRNA qPCR analyses. Quantitative PCR analysis was conducted using StepOnePlus™ (Thermo Fisher Scientific). A cycle threshold (Ct) of 40 cycles was used. The 2-ΔΔCt method was used to analyze gene expression. miR PCR Primer for hsa-miR-21-5p and cel-miR-39-3p were used (Cat. No. A25576 and 4427975, respectively, Thermo Fisher Scientific).

Western blotting

Mammalian Protein Extraction Reagent (Cat. No. 78501, Thermo Fisher Scientific) was used to extract total proteins from macrophages. The protein concentration was adjusted to 20-40 mg, separated using 4-12% sodium dodecyl sulfate-polyacrylamide gel electrophoresis, and electrophoretically transferred onto polyvinylidene fluoride membranes using an iBlot™ dry blotting system (Thermo Fisher Scientific). The membranes were then incubated for one hour with a blocking buffer (casein-free buffer for phospho-Akt: total Akt; Cat. No. 05999-84, Nacalai, Kyoto, Japan) or 5% non-fat dry milk in Tris-buffered saline with 0.5% Tween-20 (TBS-T) to block residual protein-binding sites. After overnight incubation at 4°C with each primary antibody diluted in TBS-T, the membranes were incubated with horseradish peroxidase-conjugated secondary antibodies diluted in TBS-T for one hour. Next, the membranes were exposed to enhanced chemiluminescence reagents (Cat. No. GERPN2106, GE Healthcare Life Sciences, Chicago, IL, USA) for five minutes, imaged using an Ez-Capture II system (ATTO Technology, Inc., Amherst, NY, USA), and band intensities were quantified using ImageJ software. The primary antibodies used were: anti-phospho-Akt (Thr308) (D25E6, 1:1000 dilution; Cat. No. 13038S, Cell Signaling Technology, Danvers, MA, USA), anti-total-Akt (C67E7, 1:1000 dilution; Cat. No. 4691, Cell Signaling Technology), anti-phosphatase and tensin homolog (PTEN; 138G6, 1:1000 dilution; Cat. No. 9559S, Cell Signaling Technology), anti-programmed cell death 4 (PDCD4; D29C6, 1:1000 dilution; Cat. No. 9535S, Cell Signaling Technology), and β-actin (13E5, 1:1000 dilution; Cat. No. 5125, Cell Signaling Technology). Anti-rabbit HRP-linked IgG antibody (1:8000 dilution; Cat. No. 7074, Cell Signaling Technology) was used as the secondary antibody.

Enzyme-linked immunosorbent assay (ELISA)

Interleukin-10 (IL-10) concentration in the culture medium was measured using a human IL-10 ELISA kit (Cat. No. ab185986, Abcam, Cambridge, UK) according to the manufacturer’s instructions.

MicroRNA transfection

mirVana™ miR-21 mimic and inhibitor (Cat. No. 4464066 and 4464084, respectively, Thermo Fisher Scientific) transfection was conducted using an Amaxa™ Nucleofector™ with the Human Macrophage Nucleofector Kit (transfection program Y-010) and the Cell Line Nucleofector Kit V (transfection program V-001) according to the manufacturer’s protocol (Cat. No. VPA-1008, Lonza, Basel, Switzerland). The effects of miR-21 mimic and inhibitor on phagocytosis by human macrophages and their associated intracellular pathways were compared to mirVana™ miRNA negative controls (Cat. No. 4464061 and 4464076, respectively, Thermo Fisher Scientific) after a 24-hour incubation for transfection, followed by an additional eight-hour incubation for the phagocytosis study.

Statistical analysis

Sample size estimation (N=15) for the effect of miR-21 on changes in phagocytic activity of human macrophages was performed using G*Power 3.1.9.2 (http://www.gpower.hhu.de/), with α = 0.05, β = 0.2, and effect size = 0.8. Statistical analyses were performed using GraphPad Prism 10.0.1 software (GraphPad Software, San Diego, CA, USA). The Shapiro-Wilk test was performed to determine whether the dataset followed a normal distribution. Comparisons between groups were performed using an unpaired two-tailed t-test or the Mann-Whitney U test. Comparisons among groups were conducted using two-way ANOVA with repeated measurements followed by post-hoc Bonferroni’s test or the Kruskal-Wallis test followed by post-hoc Dunn’s test. Data are expressed as the mean ± standard error of the mean or relative changes from baseline values. Statistical significance was set at p < 0.05.

## Results

LPS induces the highest *E. coli* particle phagocytosis at specific concentration and time point

**Figure 1:**
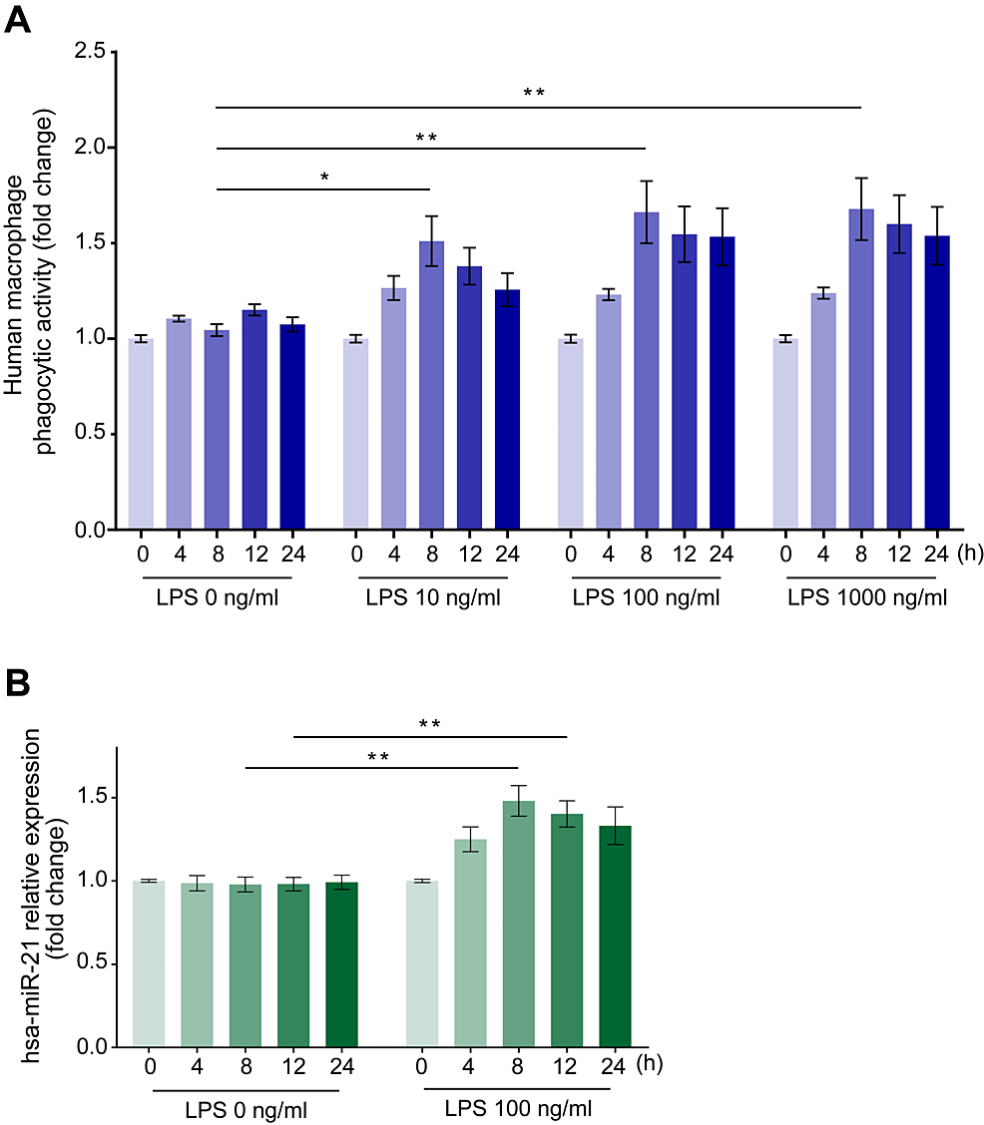
Time course changes in the phagocytic activity of human macrophages on Escherichia coli particles and miR-21 expression changes in human macrophages after stimulation with various LPS concentrations. Incubation with LPS augmented the phagocytic activity of *E. coli* particles (A) and miR-21 expression changes (B) in human macrophages at each time point from 0 to 24 hours. Data are expressed as relative changes from baseline values ± standard error of the mean (n = 15). *p < 0.05 and **p < 0.01 compared to the negative control (LPS = 0 ng/ml) at the corresponding elapsed time points (Dunn test). LPS: Lipopolysaccharide; miR-21: MicroRNA-21.

To examine the effect of LPS on phagocytosis, we cultured human macrophages with LPS (10, 100, or 1000 ng/ml for 4, 8, 12, or 24 hours) and measured their phagocytic activity on *E. coli* particles. The administration of LPS at concentrations above 100 ng/ml promoted phagocytic activity, reaching its peak of 1.69 ± 0.15-fold relative to the negative control (LPS = 0 ng/ml) after 8 hours (Figure 1A, p < 0.01).

LPS induces the highest miR-21 expression in human macrophages after eight hours

We also examined changes in miR-21 expression in human macrophages over time after incubation with 100 ng/ml LPS, observing its upregulation, which reached a peak of 1.54 ± 0.09-fold relative to the negative control (LPS = 0 ng/ml) after 8 hours (Figure 1B, p < 0.01).

LPS downregulates PTEN and PDCD4 protein expression in human macrophages after eight hours

**Figure 2:**
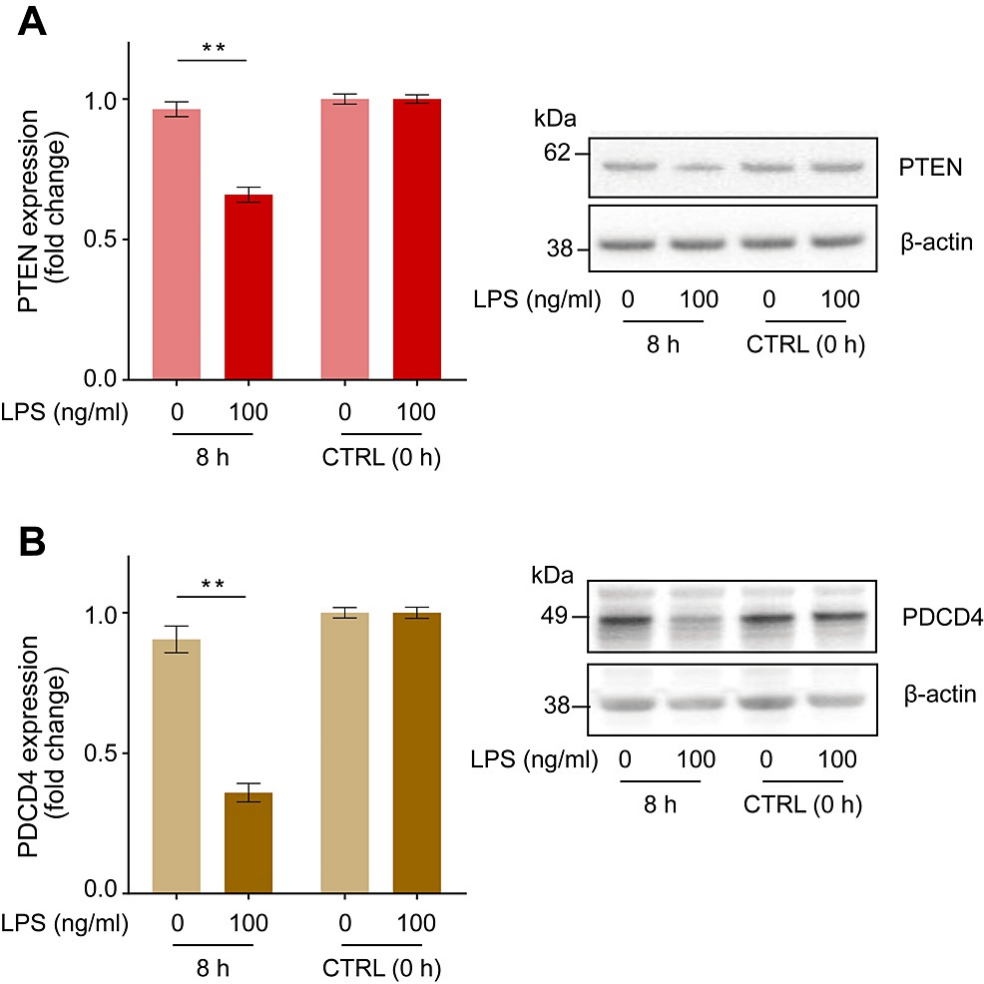
LPS-induced changes in PTEN and PDCD4 protein expression in human macrophages. Incubation with LPS (100 ng/ml) for 8 hours downregulated the expression of PTEN (A) and PDCD4 (B) in human macrophages. Representative blots are shown in the right panel. Protein levels were normalized to those of β-actin and are expressed as relative fold changes from baseline values ± standard error of the mean compared to the control (CTRL, 0 h) (n = 15). **p < 0.01 compared to the negative control (LPS = 0 ng/ml) at the corresponding elapsed time point (two-way ANOVA with repeated measurements followed by post-hoc Bonferroni’s test). PTEN: Phosphatase and tensin homolog; PDCD4: Programmed cell death 4; LPS: Lipopolysaccharide.

We examined the changes in PTEN and PDCD4 protein expression levels after 8 hours of incubation with 100 ng/ml LPS, corresponding to the maximum phagocytic activity. We observed a marked downregulation of PTEN and PDCD4 protein expression by 0.69 ± 0.03-fold (Figure 2A, p < 0.01) and 0.39 ± 0.03-fold (Figure 2B, p < 0.01), respectively, relative to the negative control (LPS = 0 ng/ml).

LPS regulates intracellular Akt phosphorylation status and IL-10 secretion in human macrophages

**Figure 3:**
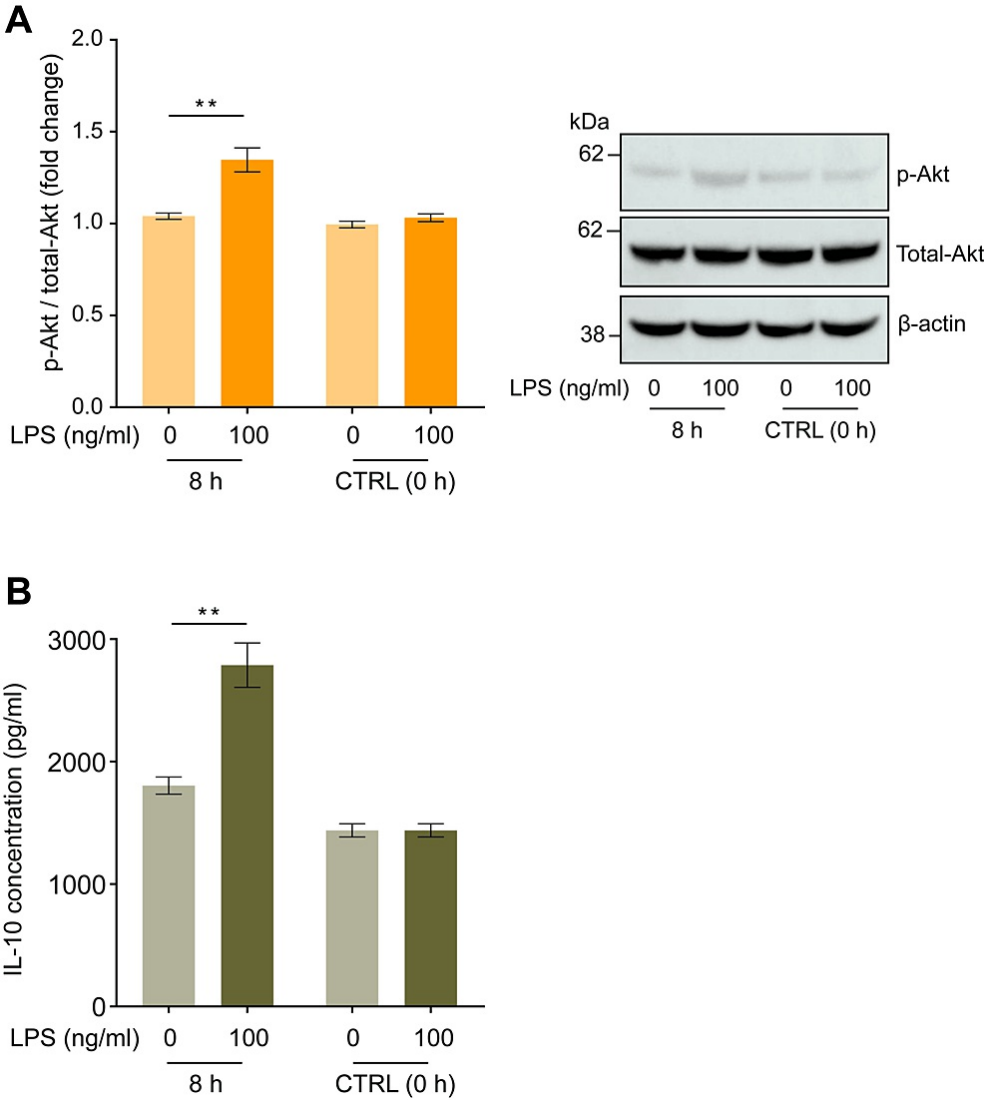
Lipopolysaccharide (LPS) induced changes in Akt phosphorylation in human macrophages and IL-10 concentrations in the culture medium. Incubation with LPS (100 ng/ml) for eight hours augmented the increase in Akt (pT308) phosphorylation in human macrophages (A) and IL-10 concentration in the cell culture medium (B). Representative blots are shown in the right panel. Protein levels were normalized to those of total Akt and are expressed as relative fold changes ± standard error of the mean relative to the control (CTRL, 0 h) (n = 15). **p < 0.01 compared to the negative control (LPS = 0 ng/ml) at the corresponding elapsed time point (Dunn test). LPS: Lipopolysaccharide.

We examined changes in the intracellular Akt phosphorylation status of human macrophages and IL-10 concentration in the culture media after incubation with LPS for 8 hours. LPS administration promoted intracellular Akt phosphorylation, increasing the ratio of phosphorylated (Thr308)/total Akt by 1.29 ± 0.06-fold (Figure 3A, p < 0.01), and increased IL-10 secretion into the culture media to 983 ± 215 pg/ml compared with the negative control (LPS = 0 ng/ml) (Figure 3B, p < 0.01).

MicroRNA transfection: enhanced and reduced expression of miR-21, respectively, increases and decreases the phagocytic activation of human macrophages

**Figure 4:**
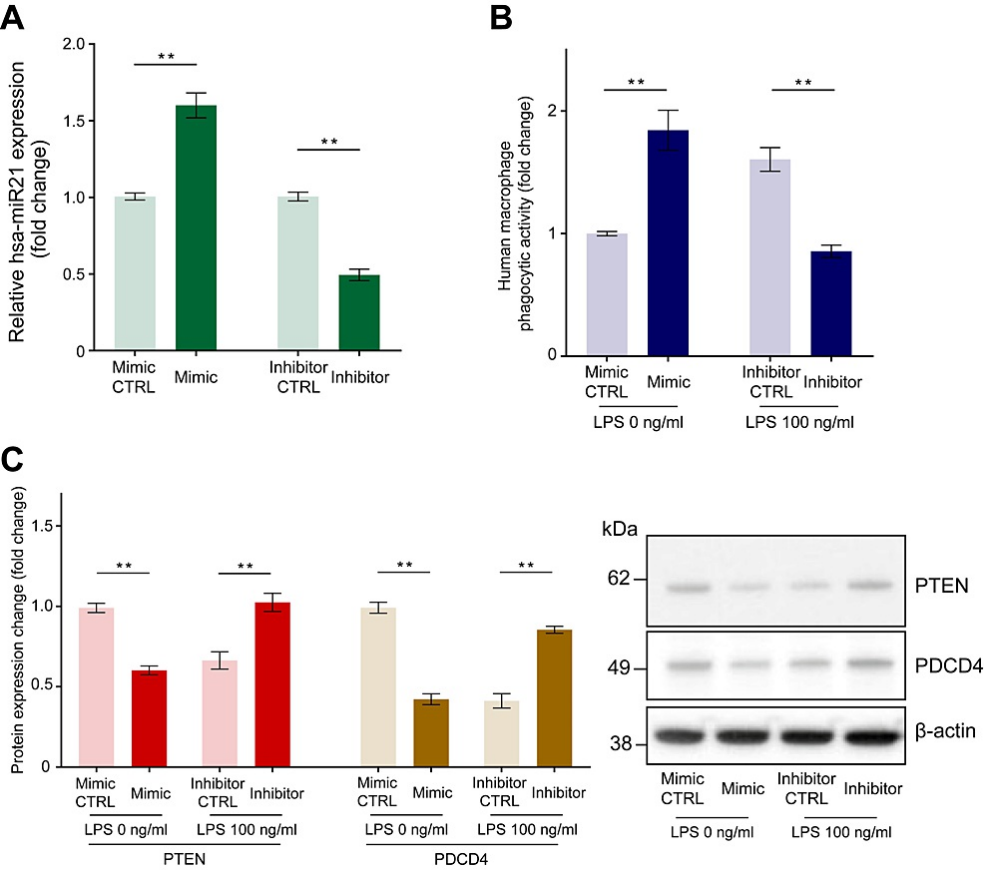
The effect of miR-21 mimic transfection and miR-21 inhibitor transfection on miR-21 expression level, phagocytic activity, and PTEN and PDCD4 expression in human macrophages. Macrophages were transfected with miR-21 mimic, miR-21 inhibitor, or negative control miRNA (CTRL). Quantitative PCR revealed increased and decreased miR-21 expression 24 hours following miR-21 mimic and inhibitor transfection, respectively (A). We conducted experiments with an 8-hour incubation of fluorescein-isothiocyanate-labeled *Escherichia coli* particles: a miR-21 mimic group without LPS and a miR-21 inhibitor group with LPS (100 ng/ml). Increased phagocytic activity of *E. coli* particles in macrophages upon transfection with a miR-21 mimic and reduced phagocytic activity after miR-21 inhibitor transfection were observed (B). A reduction in the protein levels of PTEN and PDCD4 was observed after miR-21 mimic transfection, while the opposite effect was observed after miR-21 inhibitor transfection (C). Protein levels were normalized to those of β-actin. Data are expressed as relative fold changes ± standard error of the mean relative to CTRL (n = 15). **p < 0.01 compared to each CTRL. (A: t-test; B and C: Mann–Whitney U test). LPS: Lipopolysaccharide; miR-21: MicroRNA-21.

We subsequently investigated the mechanism by which miR-21 expression modulates phagocytosis in human macrophages. Macrophages were transfected with miR-21 mimic, miR-21 inhibitor, or negative control miRNA. The qPCR analysis revealed increased and decreased miR-21 expression by 1.60 ± 0.08-fold and 0.49 ± 0.04-fold 24 hours after administration of an miR-21 mimic and miR-21 inhibitor transfection, respectively (Figure 4A, p < 0.01).

Thereafter, we conducted phagocytosis experiments using an 8-hour incubation of fluorescein-isothiocyanate-labeled *E. coli* particles. Compared to that with the negative control microRNA, transfection with the miR-21 mimic increased *E. coli* particle phagocytosis by 1.83 ± 0.15-fold after 8 hours without LPS stimulation, whereas transfection with the miR-21 inhibitor decreased the phagocytic activity by 0.55 ± 0.03-fold after incubation with LPS (100 ng/ml) for 8 hours (Figure 4B, p < 0.01).

Enhanced miR-21 expression decreases PTEN and PDCD4 expression, while reduced miR-21 expression increases their expression

To explain the reduced PTEN and PDCD4 expression observed after LPS treatment, we monitored the expression of PTEN and PDCD4 after transfection with the miR-21 mimic, inhibitor, or negative control miRNA. Compared to negative control miRNA transfection, miR-21 mimic transfection decreased the expression of PTEN and PDCD4 by 0.61 ± 0.03-fold and 0.44 ± 0.05-fold, respectively, while miR-21 inhibitor transfection increased it by 1.61 ± 0.07-fold and 2.38 ± 0.22-fold, respectively (Figure 4C, p < 0.01).

Enhanced miR-21 expression increases Akt phosphorylation and IL-10 concentrations, while reduced miR-21 expression shows the opposite

**Figure 5:**
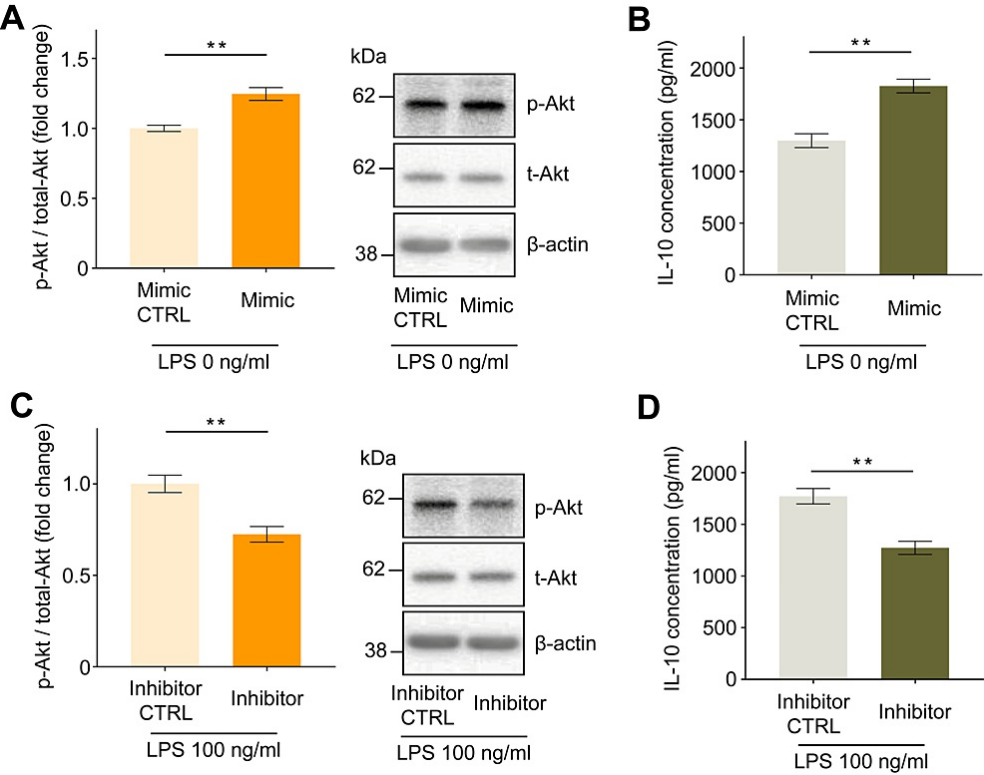
Effect of miR-21 mimic or miR-21 inhibitor transfection on the intracellular phosphorylation status of Akt and IL-10 secretion in human macrophages. Increased intracellular Akt phosphorylation (A) and IL-10 secretion into the culture medium (B) were observed following miR-21 mimic transfection. Reduced intracellular Akt phosphorylation (C) and IL-10 secretion into the culture medium (D) were observed following miR-21 inhibitor transfection. Protein levels were normalized to those of total Akt. Data are expressed as relative fold changes or pg/ml ± standard error of the mean relative to the negative control miRNA group (CTRL) (n = 15). **p < 0.01 compared to each CTRL. (A, B, and C: Mann–Whitney U test; D: t-test). miR-21: MicroRNA-21.

Compared to transfection with negative control microRNA, transfection with the miR-21 mimic increased Akt phosphorylation by 1.25 ± 0.05-fold and IL-10 concentration in the culture media to 529 ± 93 pg/ml (Figures 5A, 5B, p < 0.01), whereas transfection with the miR-21 inhibitor decreased Akt phosphorylation by 0.73 ± 0.03-fold and IL-10 concentration to 499 ± 66 pg/ml (Figures 5C-5D, p < 0.01).

## Discussion

This study highlights the significant role of miR-21 in intracellular pathways and downstream effectors that influence LPS-induced phagocytic activity in human macrophages. Phagocytosis in macrophages is complex and involves numerous signaling pathways and regulatory mechanisms. miR-21 is widely recognized for its substantial involvement in immune response pathways, particularly in regulating the expression of genes associated with inflammation and immune cell function [1]. PTEN and PDCD4 are two important regulatory proteins implicated in various cellular processes, including apoptosis, cell proliferation, and immune responses [6,7]. Their roles in phagocytosis, particularly their influence on signaling pathways in human macrophages, are of significant interest.

miR-21 expression is upregulated in response to LPS stimulation. When immune cells, such as macrophages, are exposed to LPS, certain miRNAs are upregulated as a response. However, miR-21 can downregulate the expression of inflammatory cytokines produced in response to LPS, establishing a feedback loop. This feedback helps modulate the immune response, ensuring it is effective yet does not escalate into an uncontrolled inflammatory reaction [8,9].

We demonstrated that miR-21 regulates phagocytosis by human macrophages. miR-21 directly targets PTEN, a critical tumor suppressor gene that regulates the PI3K/Akt signaling pathway [10]. By downregulating PTEN, miR-21 enhances the PI3K/Akt pathway, increasing cell survival, proliferation, and migration [7]. In immune cells, such as macrophages, the suppression of PTEN by miR-21 affects physiological functions, such as inflammation and immune surveillance. Thus, miR-21 promotes phagocytosis by increasing Akt phosphorylation in human macrophages through the inhibition of PTEN [11].

PDCD4, another miR-21 target [12], is involved in the regulation of transcription and translation and acts as a tumor suppressor by inhibiting neoplastic transformation and invasion. The miR-21-mediated downregulation of PDCD4 can lead to enhanced tumor cell growth and decreased apoptosis [6]. In the context of immune responses, reduced PDCD4 levels can affect the production of cytokines and other mediators that regulate inflammation and cellular immune function. Moreover, the relationship between PDCD4 and IL-10 involves complex regulatory mechanisms linked to immune responses and inflammation, although direct connections are not yet well-established. However, recent studies have reported a relationship between miR-21, PDCD4, and IL-10 in patients with systemic lupus erythematosus [13]. Notably, previous studies have shown that IL-10 promotes phagocytosis in human macrophages and monocytes [14,15]. Taken together, miR-21 promotes phagocytosis with increased IL-10 by inhibiting PDCD4.

In the present study, the concentration of IL-10 in the culture media increased when the cells were incubated without LPS, presumably because macrophage colony-stimulating factor (M-CSF)-differentiated macrophages were used. Macrophages can be polarized into pro-inflammatory (M1) and anti-inflammatory (M2) cells by granulocyte-macrophage colony-stimulating factor (GM-CSF) and M-CSF, respectively. Unlike GM-CSF, M-CSF is a ubiquitous cytokine that circulates throughout the human body [16]. Thus, M-CSF may be the primary cytokine guiding macrophage differentiation under normal conditions. M-CSF-driven macrophages (M2) have intrinsic anti-inflammatory properties and are characterized by high IL-10 production [17].

Macrophage phagocytosis is a fundamental process in the immune system. Disruption of macrophage phagocytosis can significantly affect the progression and severity of various diseases. Understanding the specific mechanisms by which miR-21 modulates phagocytosis could lead to new therapeutic strategies for diseases in which phagocytosis is dysregulated, such as chronic autoimmune diseases, cancers, neurodegenerative diseases, atherosclerosis, chronic obstructive pulmonary disease, and acute infectious diseases [18,19].

**Figure 6:**
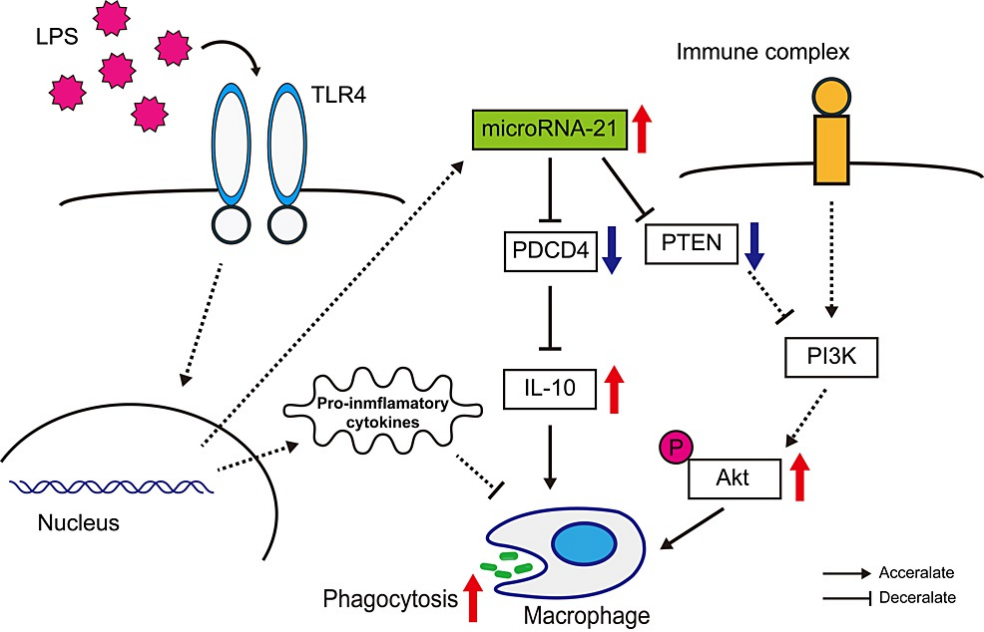
A hypothetical model for the role of miR-21 in regulating intracellular pathways involved in phagocytosis within human macrophages. This scheme illustrates that miR-21 expression is upregulated in response to LPS stimulation, leading to decreased expression of PTEN and PDCD4. These changes subsequently enhance Akt phosphorylation and increase IL-10 production, thereby activating phagocytic activity in macrophages. This image was created by the authors of this study, Yoshinobu Nakayama and Yasufumi Nakajima. LPS: Lipopolysaccharide; miR-21: MicroRNA-21; PTEN: Phosphatase and tensin homolog; PDCD4: Programmed cell death 4.

We present a detailed hypothetical model that depicts the regulatory role of miR-21 in intracellular signaling pathways associated with phagocytosis in human macrophages. This model provides insight into the molecular mechanisms by which miR-21 influences cellular processes and immune responses (Figure 6).

This study has some limitations. First, it remains unclear whether the same findings would occur in vivo. Second, the relationship between miRNAs and anti-inflammatory processes is complex and substantial, highlighting the intricacies of miRNA function within biological systems. A potential second phase of research could involve a comprehensive analysis of miRNAs using next-generation sequencing technologies, including an examination of their interactions with other miRNAs.

## Conclusions

The resolution of inflammation by macrophages is critical for restoring tissue integrity, preventing chronic illnesses, ensuring effective healing, and maintaining a well-balanced and regulated immune response. miR-21 is a well-studied miRNA that plays a significant role in various physiological and pathological processes, including inflammation. This in vitro study highlights the role of miR-21 in regulating phagocytosis in human macrophages and elucidates the associated intracellular pathways. These properties highlight the potential of miR-21 as a target for therapeutic and diagnostic strategies to control excessive inflammatory responses in various diseases.
